# Timing within the menstrual cycle, sex, and the use of oral contraceptives determine adrenergic suppression of NK cell activity

**DOI:** 10.1054/bjoc.2000.1490

**Published:** 2000-12

**Authors:** K Shakhar, G Shakhar, E Rosenne, S Ben-Eliyahu

**Affiliations:** Department of Psychology, Tel Aviv University, Tel Aviv, 69978, Israel

**Keywords:** menstrual cycle, natural killer, catecholamines, cancer, immunity

## Abstract

Physiological responses that involve adrenergic mechanisms, such as stress-induced changes in cardiovascular indices, were reported to fluctuate along the menstrual cycle. Metastatic development following surgery was also reported to vary according to the menstrual phase during which a primary breast tumour was removed. Natural killer (NK) cells are believed to play an important role in controlling metastases. Our recent studies in rats demonstrated that adrenergic suppression of NK activity and of resistance to metastasis is more profound during oestrous phases characterized by high levels of oestradiol. In the current study in humans, we examined the in vitro impact of a β-adrenergic agonist, metaproterenol (MP), on NK activity, comparing blood drawn from (a) women tested at 3–4 different phases of their menstrual cycle (*n* = 10), (b) women using oral contraceptives (OC) (*n* = 10), and (c) men (*n* = 7). NK activity in each blood sample was assessed in the presence of 5 different concentrations of MP (10^–8^M to 10^–6^M), and in its absence (baseline). The results indicated marked group differences in the magnitude of NK suppression by MP: EC _50_ was 2.6-fold lower in the luteal phase compared to the follicular phase, and 1.8-fold lower in OC users compared to men, who were least susceptible to the effects of MP. No significant group differences or menstrual effects in baseline levels of NK activity were evident. These findings provide the first empirical evidence for menstrual regulation of adrenergic impact on cellular immune competence. Relevance of these findings to the relation between the timing of breast cancer excision within the menstrual cycle and survival rates is discussed. © 2000 Cancer Research Campaign http://www.bjcancer.com


*British Journal of Cancer* (2000) **83**(12), 1630–1636
© 2000 Cancer Research Campaign
doi: 10.1054/ bjoc.2000.1490, available online at http://www.idealibrary.com on IDEAL®

http://www.bjcancer.com

### Timing within the menstrual cycle, sex, and the use of oral contraceptives determine adrenergic suppression of NK cell activity

**K Shakhar, G Shakhar, E Rosenne and S Ben-Eliyahu**

Department of Psychology, Tel Aviv University, Tel Aviv 69978, Israel

**Summary** Physiological responses that involve adrenergic mechanisms, such as stress-induced changes in cardiovascular indices, were reported to fluctuate along the menstrual cycle. Metastatic development following surgery was also reported to vary according to the menstrual phase during which a primary breast tumour was removed. Natural killer (NK) cells are believed to play an important role in controlling metastases. Our recent studies in rats demonstrated that adrenergic suppression of NK activity and of resistance to metastasis is more profound during oestrous phases characterized by high levels of oestradiol. In the current study in humans, we examined the in vitro impact of a β-adrenergic agonist, metaproterenol (MP), on NK activity, comparing blood drawn from (a) women tested at 3–4 different phases of their menstrual cycle ($n$ = 10), (b) women using oral contraceptives (OC) ($n$ = 10), and (c) men ($n$ = 7). NK activity in each blood sample was assessed in the presence of 5 different concentrations of MP ($10^{-8}$ M to $10^{-6}$ M), and in its absence (baseline). The results indicated marked group differences in the magnitude of NK suppression by MP: $EC_{50}$ was 2.6-fold lower in the luteal phase compared to the follicular phase, and 1.8-fold lower in OC users compared to men, who were least susceptible to the effects of MP. No significant group differences or menstrual effects in baseline levels of NK activity were evident. These findings provide the first empirical evidence for menstrual regulation of adrenergic impact on cellular immune competence. Relevance of these findings to the relation between the timing of breast cancer excision within the menstrual cycle and survival rates is discussed. © 2000 Cancer Research Campaign http://www.bjcancer.com

**Keywords:** menstrual cycle; natural killer; catecholamines; cancer; immunity

Several studies have demonstrated that the menstrual cycle modulates the intensity of adrenergic responses as well as the severity of several pathological conditions that involve adrenergic mechanisms. For example, increased cardiovascular responses to stress were reported to occur during the luteal phase of the menstrual cycle (Manhem et al, 1991; Tersman et al, 1991), and the administration of oestrogen potentiated the stress-induced increase in heart rate and blood pressure (Manhem et al, 1996). Asthma, another adrenergic-mediated condition, was reported to worsen during the premenstrual period (Pauli et al, 1989; Case and Reid, 1998), and treatment with progesterone was reported to prevent menstrually related exacerbation of asthma attacks (Beynon et al, 1988). Additionally, about 60% of women suffering from migraine headaches experience an increase in the frequency of migraines during the perimenstrual phase (Case and Reid, 1998). Migraines may also involve adrenergic processes, as plasma levels of noradrenaline decrease and its metabolite levels increase during attacks (Marcus, 1995).

Although ample evidence indicate that stress and adrenergic activity can affect immune competence (Sheridan et al, 1994; Kiecolt-Glaser and Glaser, 1995; Benschop et al, 1996a; Cohen and Herbert, 1996; Shakhar and Ben-Eliyahu, 1998), little is known about possible modulation of such effects by the menstrual cycle. Wheeldon et al (1994) reported that $\beta_2$-adrenoceptor density and cAMP generation in response to isoproterenol in lymphocytes are greater during the luteal phase compared to the follicular phase. Other researchers, on the other hand, recorded no menstrual variations in these indices (Santala et al, 1990; Litschauer et al, 1998). Administration of progesterone during the follicular phase upregulated the expression of $\beta_2$-adrenoceptor by lymphocytes (Tan et al, 1996), suggesting the involvement of a specific sex steroid. Redistribution of WBC subpopulations (NK cells, CD8 cells, CD3 cells and CD4 cells) following brief stress, was reported to be unaffected by the menstrual cycle (Mills et al, 1995).

An immune function markedly affected by stress in both animals and humans, is natural killer (NK) cell activity (Irwin, 1993; Schedlowski et al, 1993; Bachen et al, 1995; Ben- Eliyahu et al, 1999; Laudenslager et al, 1999). NK cells are large granular lymphocytes that lyse virally infected cells and malignant cells. Catecholamines have been implicated in mediating the effects of stress on NK activity (Benschop et al, 1996b; Shimizu et al, 1996; Klokker et al, 1997; Ben-Eliyahu, 1998b). Human NK cells express $\beta_2$, $\alpha_1$ and $\alpha_2$ adrenoceptors (Jetschmann et al, 1997), and β-adrenergic agonists were shown to suppress NK activity in vitro by elevating intracellular cAMP levels (Whalen and Bankhurst, 1990). Recent studies in rats have shown that in-vivo administration of β-adrenergic agonists suppressed NK activity in a dose-dependent manner, consequently compromising host resistance to metastasis (Shakhar and Ben-Eliyahu, 1998).

Studies that examined baseline levels of human NK activity during different phases of the menstrual cycle yielded inconsistent

*Received 2 May 2000*
*Revised 25 July 2000*
*Accepted 9 August 2000*

*Correspondence to:* S Ben-Eliyahu results. Some reported a decrease in NK activity during the follicular phase (White et al, 1982), some a significant reduction around the periovulatory period (Sulke et al, 1985), and others reported no differences (Thyss et al, 1984). Animal studies are also inconsistent in this respect (Hrushesky et al, 1988; Ben-Eliyahu et al, 1996). It is our hypothesis that whereas baseline levels of NK activity may not be affected by the ovulatory cycle, the suppressive effects of stress, specifically those induced by catecholamines, are modulated by the ovulatory cycle. This hypothesis is in line with the above-mentioned alterations in adrenoceptors expression and cAMP responsivity along the menstrual cycle, and the major role they play in suppressing NK activity under stress conditions.

Our recent studies in rats have supported this hypothesis: whereas baseline levels of NK activity did not fluctuate along the oestrous cycle, the in-vitro suppression of NK activity by a β-adrenergic agonist was more pronounced in blood drawn during the proestrus and oestrus phases (Ben-Eliyahu et al, 2000). Corresponding findings were obtained using an NK-sensitive model of metastasis. Administration of a β-adrenergic agonist more profoundly suppressed resistance to metastasis during the same cycle phases. Therefore, in the present study we examined whether the menstrual cycle would affect the sensitivity of human NK cells to β-adrenoceptor stimulation. Blood samples were taken from women at different phases of the menstrual cycle, women using oral contraceptives, and men, and both baseline NK activity and the in-vitro response of NK cells to a β-adrenergic agonist were assessed.

### MATERIALS AND METHODS

### Subjects

Overall 27 subjects, 21–52 years old (mean = 25.5), were recruited to participate in the study. The aims of the study and its specific protocol were explained to all subjects. All participants gave their informed consent prior to the experiment. 11 subjects were women who had a regular menstrual cycle (25–32 days) for at least 6 months prior to the experiment (mean age 29.9 ± 11.6), 9 subjects have been using oral contraceptives (OC) for at least 6 months (mean age 22.1 ± 1.4), and 7 subjects were men (mean age 23.6 ± 2.06). Whenever these 3 groups were compared, the 4 oldest women with a regular menstrual cycle were excluded from the analysis to obtain age-matched groups (reducing the mean age in this group to 21.3 ± 0.5). All subjects filled a medical questionnaire and reported being free of acute or chronic disease and substance abuse. Smokers were evenly distributed among the 3 groups (1 or 2 in each group). The study was approved by the Ethics committee of Tel-Aviv University and conformed to the Helsinki Declaration.

### Experimental procedure

The experiment was conducted over 4 sessions during 4 consecutive weeks. Each woman with a regular cycle was tested at 3–4 different sessions spanning her menstrual cycle. In each session 2 men, 2–3 women who use OC and 7–10 women with a regular cycle participated. Blood was collected into 1 non-heparinized and 3 heparinized (50 U $ml^{-1}$ blood) 12 ml vacuum test tubes between 07:30 and 09:00 a.m. The order of blood collection was randomized between the different experimental groups, and the experimenters were blind to the origin of blood samples. Whole and washed blood NK cytotoxicity assays were performed on the freshly drawn blood immediately after blood collection.

Aliquots of each blood sample were tested for NK activity in the presence of different concentrations of the β-agonist metaproterenol (MP), or in its absence (baseline). Aliquots of the non-heparinized blood were used to assess levels of sex hormones from each blood sample.

### Determination of menstrual phase

The menstrual phase at blood collection was determined based on serum levels of oestradiol, progesterone, and LH measured in each blood sample, combined with the women's report of the dates of the menses preceding and following each session. In order to describe the changes along the menstrual cycle in details, it was divided into 7 phases (see Figure 4 for hormonal levels). Because each woman was tested no more than 4 times, this division could not have been used for repeated measure analysis. For this purpose, the menstrual cycle was divided to follicular and luteal phases. The follicular phase included an interval of up to 12 days following the first day of menses, and the luteal phase included the 11 days preceding the first day of menses. This division was done according to the above-mentioned criteria. Hormonal levels and self report of menses closely agreed in all cases except in one subject that was thus excluded from the study. 3 blood samples taken around the ovulation phase were not included in this analysis. Radioimmunoassay kits (Diagnostic Products Corp, Los Angeles, CA) were used to measure serum levels of the three hormones.

### Oral contraceptives

Most subjects used monophasic OC with 0.03 mg ethinyl oestradiol (EE) combined with either 0.075 mg gestodene ($n$ = 5), 0.25 mg norgestimate ($n$ = 1), or 0.15 mg desogestrel ($n$ = 2). One subject took a monophasic OC with 0.035 mg EE and 2 mg cyproterone acetate, and 1 subject took triphasic OC with the following dosage: 6 tablets with 0.03 mg EE + 0.05 mg levonorgestrel, 5 tablets with 0.040 mg EE + 0.075 mg levonorgestrel and 10 tablets with 0.03 mg EE + 0.125 mg levonorgestrel.

### Metaproterenol

(MP) (orciprenaline) (Sigma, Israel) is a non-selective β-adrenergic agonist, with a higher affinity to $\beta_2$ receptors than to $\beta_1$ (Dengler and Hengstmann, 1976). MP was dissolved on the morning of each experiment in phosphate buffered saline (PBS).

### Radiolabelling of K562 target cells

The standard NK-sensitive K562 erythromyeloid tumour line (Lozzio and Lozzio, 1979) was used as the target for the NK cytotoxicity assay. The cells were grown in complete medium (CM) (RPMI 1640 medium supplemented with 10% heat-inactivated fetal calf serum (FCS), 50 μg $ml^{-1}$ gentamycin, 2 mM L-glutamine, 0.1 mM non-essential amino acids, and 1 mM sodium pyruvate (Biological industries, Beit Haemek, Israel)) at 37°C in 5% $CO_2$. For radiolabelling, cells were incubated for 1 hour with 200 μCi $^{51}Cr$, 400 μl FCS, and 300 μl CM per $4 \times 10^7$ cells. Following incubation, cells were washed twice

© 2000 Cancer Research Campaign 

with CM (300 ***g***, for 10 min) and adjusted to the desirable concentrations.

### NK cytotoxicity assay

Two different procedures were used to assess NK activity, the washed blood assay, in which the serum is replaced with CM, and the whole blood assay, in which the subject's serum is preserved. Both procedures differ from the standard NK assay in that they assess anti-tumour cytotoxicity following minimal manipulation on the blood (e.g., no exclusion of cell populations or adhesion sorting), and are completed within a very short period after blood withdrawal. The procedures were previously validated and used in humans, and were shown to reflect cytotoxicity carried out by NK cells, and not by other cell types or serum factors (Ottenhof et al, 1981; Ree and Platts, 1983). The 6 different concentrations of target cells (K562) used in each procedure were determined in pilot studies and set to achieve optimal levels of killing (increasing toward a plateau). Possible direct effects of MP on the K562 target cells were assessed to distinguish between the effects of MP on NK activity and its effect on target cells. As in our previous animal studies (Ben-Eliyahu et al, submitted; Giboney and Ben-Eliyahu, 2000), no direct effects on target cells were evident.

### *Washed blood assay*

For each subject, 12 ml of blood were washed once with PBS and once with CM. In each wash, blood was diluted 3-fold in either PBS or CM, centrifuged at 400 ***g*** for 10 min, and the supernatant was removed to restore the original blood volume. NK activity was tested against 6 concentrations of target cells and in the presence of different concentrations of MP ($10^{-6}$ M, $3 \times 10^{-7}$ M, $10^{-7}$ M, $3 \times 10^{-8}$ M, $10^{-8}$ M) and, in duplicates, in its absence (Baseline). 200 µl of blood were placed in each well of a microtitre plate, and 50 µl of the $^{51}$Cr-labelled K562 tumour cells in CM containing one concentration of MP, were then added on top of the blood. A concentration of 100 000 K562 cells per well was used, and consecutively diluted by two for lower concentrations. To determine spontaneous and maximal $^{51}$Cr release, blood was substituted with 200 µl of CM or HCl (4%), respectively. Plates were centrifuged at 500 ***g*** for 10 min to create a buffy coat layer of leucocytes and target cells on top of the red blood cells, and were then incubated for 4 hours in 5% $CO_2$ at 37˚C. Following incubation, plates were centrifuged again and aliquots of 100 µl of the supernatant were recovered from each well for assessment of radioactivity in a γ-counter.

### *Whole blood assay*

The exact same steps as in the washed blood procedure were followed without 'washing' the blood, i.e. cytotoxicity was assessed in the presence of autologous serum.

### Data analysis

### *Percent specific lysis by NK cells*

Percent specific lysis for each target cell concentration was calculated using the standard formula below (Ottenhof et al, 1981).

$$\frac{(\mathrm{CPM} - \mathrm{b}) \times \dfrac{\mathrm{Vt} - (\mathrm{Vb} \times \mathrm{Hct})}{\mathrm{Vt}} - (\mathrm{SR} - \mathrm{b})}{(\mathrm{MR} - \mathrm{b}) - (\mathrm{SR} - \mathrm{b})}$$

CPM – count per min; SR – $^{51}$Cr spontaneous release per min; MR – $^{51}$Cr maximum release per min; b – background count per min; Vt – total volume in well (blood + target cells); Vb – total blood volume in well; Hct – % hematocrit.

Since the $^{51}$Cr released by the labelled target cells is found only in the supernatant above the red blood cells (Ottenhof et al, 1981), the total volume is corrected by subtracting the red blood cell volume (% haematocrit (Hct) × blood volume (Vb)) from the total volume (Vt).

### *Lytic units and sensitivity of NK activity to MP*

The purpose of this procedure was to assess the sensitivity of NK activity to adrenergic stimulation regardless of the baseline activity levels (lysis at the absence of MP). Noteworthy, these baseline levels did not differ between the groups. First, for each blood sample, we used baseline NK activity to determine the

$$1/3 \text{ increment} = \frac{(\mathrm{Max}_{\mathrm{Baseline}} - \mathrm{Min}_{\mathrm{Baseline}})}{3}$$

percent specific lysis at which one third of the increment in lysis is achieved. The formula used was:

where $\mathrm{Min}_{\mathrm{Baseline}}$ is the minimal level of lysis and $\mathrm{Max}_{\mathrm{Baseline}}$ is the maximal level of lysis. Next, Lytic Units 1/3 ($LU_{1/3}$) were defined as the concentration of target cells in which this level was achieved in all conditions studied within this blood sample. Particularly, the regression exponential fit method (Pollock et al, 1990) was used to infer $LU_{1/3}$ from the empirical data (of percent specific lysis at the six E:T ratios), in respect to baseline levels as well as to each concentration of MP.

Next, to represent the extent of NK suppression at different concentrations of MP we used the formula: $LU_{MPx}/LU_{Baseline}$ (based on Friberg et al, 1996) where $LU_{MPx}$ is $LU_{1/3}$ at x concentration of MP, and $LU_{Baseline}$ is $LU_{1/3}$ in the absence of MP (baseline).

Finally, to represent the sensitivity of NK activity at different menstrual phases and in different groups we calculated Effective $\mathrm{Concentration}_{50}$ ($EC_{50}$) – the concentration of MP needed to suppress NK activity to 50% of baseline levels, i.e., the concentration in which $LU_{MPx}/LU_{Baseline} = 0.5$.

### Statistical analysis

For statistical analysis either factorial ANOVA or within-subject repeated measures ANOVA were used. Provided significant group differences existed, Scheffé post-hoc analyses were conducted. Alpha level was set to 0.05 for all analyses.

Data from both the whole blood and washed blood assays were analysed and correlations between the two were computed.

To compare baseline levels of the different groups, percent specific lysis was used. The data from the 4 different sessions were combined based on average baseline levels of men and OC users.

Whenever NK activity was compared between groups (i.e. women with a regular cycle, OC users, and men), the 4 oldest subjects of women with a regular menstrual cycle were removed in order to age-match the groups. Additionally, the data of women with a regular menstrual cycle, who participated in several sessions, were averaged across the menstrual cycle. Similarly, whenever the luteal and follicular phases were compared, the average of the indices in each phase was used.

*© 2000 Cancer Research Campaign*

### RESULTS

### Correlations between percent specific lysis in the whole blood and washed blood assays

The Baseline Percent Specific Lysis of each subject in the washed blood and whole blood assays were highly correlated (r = 0.88, $P < 0.05$). In the different levels of MP, the correlation ranged from 0.83 to 0.89.

Since in most cases the results in the whole and washed blood assays were similar, only figures of the washed blood results are presented. Each figure represents the combined data of the four experimental sessions conducted.

### Suppression of NK activity by *β*-adrenergic stimulation

When added in vitro to blood samples, MP caused a dose-dependent decrease in NK activity (Figure 1). This suppression was evident in all subjects. Trend analysis using concentration of MP and concentration of target cells as repeated measures, indicated a significant linear dose dependency both in the washed blood ($F(1, 26) = 291.20$, $P < 0.0001$) and in the whole blood assays ($F(1, 26) = 218$, $P < 0.0001$).

### Baseline levels of NK activity: the effects of sex, use of OC and the menstrual cycle

No significant difference was found in baseline NK activity between the follicular and the luteal phases (ANOVA with both E:T ratio and the cycle phase as repeated measures). No significant differences in Baseline levels of NK cytotoxicity per ml of blood were revealed among the three groups (i.e. women with a regular menstrual cycle, OC, men) (ANOVA with E:T ratio as a repeated measure).

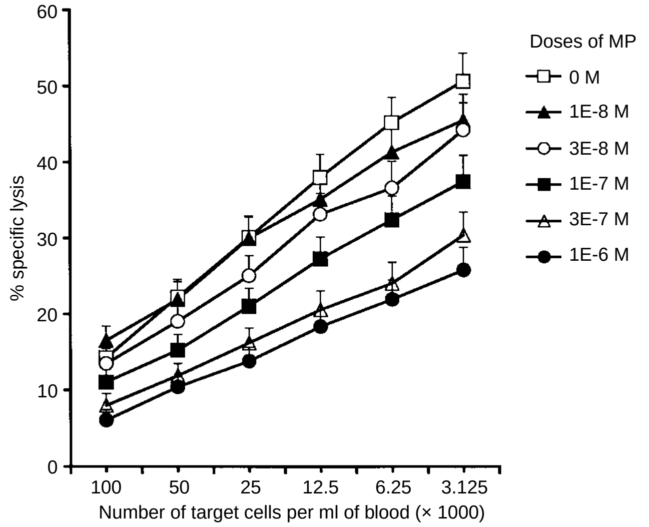

**Figure 1** The effect of increasing concentrations of the β-adrenergic agonist metaproterenol (MP) on blood NK activity (mean ± SEM) at different effector to target (E:T) ratios. In vitro exposure to MP suppressed cytotoxicity in a dose-dependent manner

### Sensitivity of NK activity to *β*-adrenergic stimulation: the effects of sex, use of OC and the menstrual cycle

NK activity was markedly more suppressed by MP during the luteal phase than during the follicular phase, both in the washed blood assay and the whole blood assay. By and large, during the follicular phase, 3-fold higher concentrations of MP were needed to achieve the same level of suppression as during the luteal

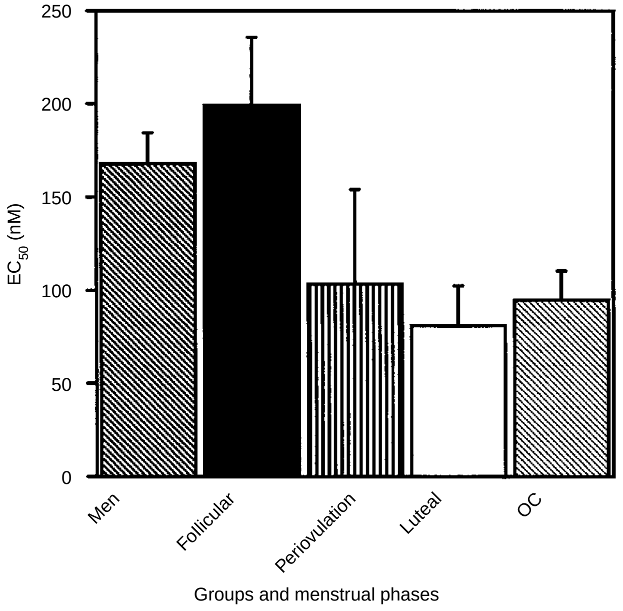

**Figure 2** The suppressive effect of MP on NK activity in men, oral contraceptive users (OC), and in women with a regular cycle. Blood samples from women in the follicular phase and from men needed higher concentrations of MP to induce 50% suppression from baseline levels ($EC_{50}$). The statistical analysis for comparing the follicular and luteal phases was conducted using a repeated measure ANOVA, while the comparison among the three groups (men, OC users, and women with a regular cycle) was conducted separately using a factorial ANOVA (the data from each woman with a regular cycle was averaged across the cycle)

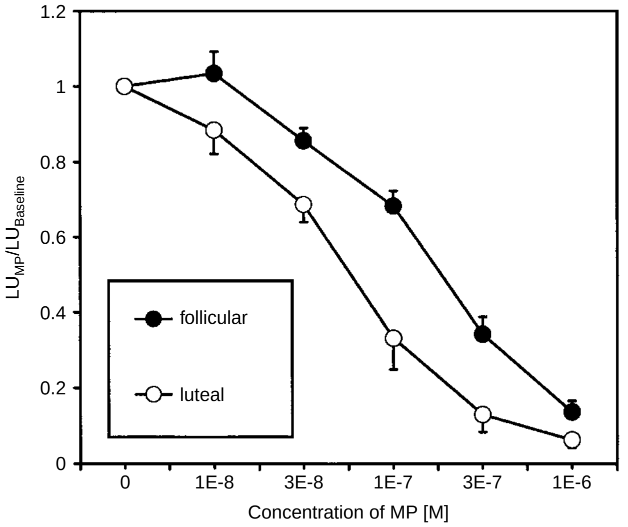

**Figure 3** Sensitivity of NK activity to different doses of MP during the follicular and luteal phases of the menstrual cycle. NK sensitivity is expressed as the ratio between lytic units with MP and lytic units in the absence of MP (mean ± SEM). During the follicular phase a 3-fold higher concentration of MP was needed to reach the same levels of suppression as in the luteal phase

© 2000 Cancer Research Campaign

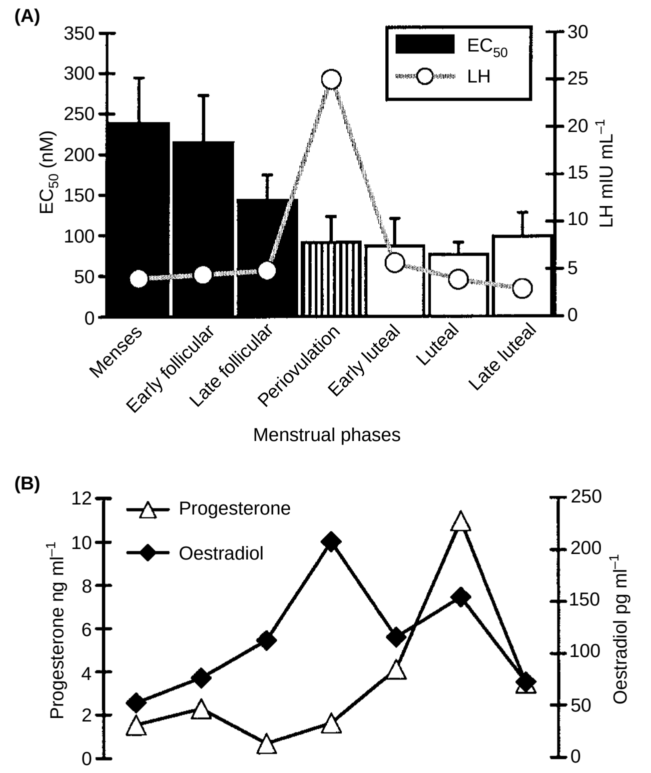

**Figure 4** NK sensitivity to MP along 7 phases of the menstrual cycle ($EC_{50}$) (bars, mean ± SEM) (**A**). NK sensitivity is expressed in $EC_{50}$ (MP concentration needed to induce 50% suppression from baseline level). Shown in lines are the serum levels of LH, oestradiol and progesterone assessed in these women along the menstrual cycle **(A & B)**

phase (Figure 3). Two way repeated measure ANOVA indicated a significant main effect of the menstrual cycle in both the washed blood assay ($F (1, 8) = 12.36$, $P < 0.01$) and the whole blood assay ($F (1, 8) = 9.713$, $P < 0.05$) ($LU_{MPx}/LU_{control}$ was used as the dependent variable). As indicated above, MP had a significant main effect.

Changes in $EC_{50}$ throughout the seven phases of the menstrual cycle are presented in Figure 4. No statistical analysis is available due to many missing values.

When comparing the 3 groups, significant differences in $EC_{50}$ were found using an ANOVA in the washed blood assay ($F (2, 22) = 4.33$, $P < 0.05$) but not in the whole blood assay. A post-hoc analysis showed that men had 75% higher $EC_{50}$ than OC users ($P < 0.05$) (Figure 2).

### DISCUSSION

This study examined the effects of the menstrual cycle, sex, and the use of oral contraceptives on baseline levels of NK activity and on the sensitivity of NK cells to β-adrenoceptor stimulation. As hypothesized, alteration in NK activity along the menstrual cycle occurred only under conditions of β-adrenoceptor stimulation. Specifically, suppression of NK activity by β-adrenoceptor stimulation was significantly greater during the luteal phase than during the follicular phase. The suppression of NK activity in OC users was similar to the marked suppression evident during the luteal phase, whereas the suppression in men was similar to the lower levels of suppression evident during the follicular phase. These effects were evident using either methods of assessing NK activity: the washed blood method, which resembles standard procedures, and the whole blood method, which preserves autologous serum. On the other hand, no differences in baseline levels of NK activity were evident between menstrual phases or among the three experimental groups (men, OC users and women with a regular cycle).

Potential sex hormones underlying these effects have yet to be identified. Both oestradiol and progesterone levels are higher during the luteal phase, which was characterized by greater adrenergic suppression of NK activity. Nevertheless, there is indication that oestradiol, more than progesterone, contributes to the effects of the menstrual cycle. The degree of NK sensitivity to β-adrenergic stimulation mirrored plasma levels of oestradiol better than plasma levels of progesterone (Figure 4). Our recent studies in rats, which employed a paradigm similar to the one used in the present study, further supported this suggestion. In the Fischer-344 rat, oestradiol and progesterone levels are dissociated: oestradiol and progesterone peak on proestrus phase, but progesterone has an additional peak on metoestrus phase. Our findings indicated that the high oestradiol phase, but not the high progesterone phases, was associated with a greater adrenergic suppression of NK activity (Ben-Eliyahu et al, 2000).

In agreement with the hypothesized role of female sex hormones, OC users, who are continuously exposed to analogues of sex steroids, showed suppression of NK activity that was as marked as in women during the luteal phase (which is characterized by high endogenous levels of oestradiol and progesterone). Men were as resistant to this suppression as women during the follicular phase. Nevertheless, because the groups differ in various factors other than levels of ovarian sex steroids, it is unclear whether these steroids play a significant role in determining the degree of NK sensitivity in men and OC users. Clearly, more direct evidence in humans is needed to identify potential hormonal mediators. A comparison of different regimens of replacement therapy in postmenopausal women, or the administration of specific sex hormones during the early follicular phase, could clarify this issue.

Possible cellular mechanisms underlying the modulatory effect of the menstrual cycle are alterations in β-adrenoceptor expression by NK cells, or alteration in receptor reactivity to β-adrenoceptor stimulation. Indeed, some studies reported that the luteal phase is characterized by higher expression of $\beta_2$-adrenoceptors on lymphocytes, and by a higher lymphocyte cAMP response to isoproterenol (Wheeldon et al, 1994). While NK cells comprise only about 15% of the lymphocyte population, they were reported to express the highest levels of $\beta_2$-adrenoceptors (Landmann, 1992). Activation of these receptors was shown to suppress NK activity by increasing cAMP levels (Whalen and Bankhurst, 1990). If indeed the above alterations in lymphocyte expression of $\beta_2$-adrenoceptors occur in NK cells, they may explain the effects of the menstrual cycle at the level of NK cell. If they occur on other lymphocytes, they could mediate an indirect effect on NK activity.

In respect to variations in baseline levels of NK activity along the menstrual cycle, the literature is scant and inconsistent (White et al, 1982; Thyss et al, 1984; Sulke et al, 1985). In a recent study, we have observed no differences in the number or in the activity of NK cells along the menstrual cycle (Yovel et al, 1998). The current study indicates that differences in NK activity occur under β-adrenoceptor stimulation, but not in its absence. This may suggest that the conflicting reports regarding baseline conditions could be attributed to different levels of catecholamines which are released due to stress around blood withdrawal. Indeed, in a recent study in rats, we demonstrated that the oestrous cycle modulated level of

*© 2000 Cancer Research Campaign*

NK activity following stress, but not in blood drawn from control rats when procedural stress was minimized (Ben-Eliyahu et al, 2000). Whether such stress effects persist or dissipate may depend on the delay between blood withdrawal and the assessment of NK activity.

Although our findings indicate that the ovarian cycle modulates NK sensitivity to β-adrenoceptor stimulation, the biological significance of such effects is unclear. In rats, however, we have recently found that in-vivo suppression of resistance to metastasis of a mammary tumour by a β-adrenergic agonist, as well as the in-vitro suppression of NK activity by MP, were more profound during the proestrus/oestrus phase (Ben-Eliyahu et al, submitted). These parallel effects suggest possible clinical circumstances in which the current findings in humans may prove clinically significant, i.e. resisting metastasis when catecholamine levels are high. Indeed, a major incentive for conducting our study was the controversial clinical reports that the menstrual phase during which breast tumours are removed influences long-term rates of metastatic development and survival (for review see Hagen and Hrushesky, 1998). Unfortunately, different clinical reports implicated different menstrual phases as being the high-risk period (Hagen and Hrushesky, 1998), suggesting that hospital variation in perioperative routines may determine the timing of increased susceptibility to metastases and whether it occurs. Nevertheless, whenever this clinical phenomenon was observed, it occurred only in women having positive lymph nodes at the time of surgery, indicating that the metastatic process had already begun. Because the menstrual cycle influenced survival whether or not the excised tumour expressed receptors for sex steroids, direct effects of sex hormones on malignant cells are unlikely. It is our hypothesis that the surgery itself and the psychological and physiological stress accompanying it, markedly facilitated the metastatic processes, and that certain aspects of the menstrual cycle further modulated patient resistance to metastasis in this vulnerable condition. We also suggest that adrenergic suppression of NK activity may be a mechanism contributing to the adverse effects of surgery. Suppression of NK activity after surgery is well documented clinically (Pollock et al, 1991; Brittenden et al, 1996), was attributed by animal studies to adrenergic mechanisms (Ben-Eliyahu, 1998a), and was suggested by human and animal studies to promote metastasis (Zoller et al, 1989; Pollock et al, 1991; Taketomi et al, 1998; Ben-Eliyahu et al, 1999). The current study demonstrated that such adrenergic suppression is also modulated by the menstrual cycle, thus proposing this modulation as a mechanism contributing to the clinical phenomena.

### ACKNOWLEDGEMENTS

This research was supported by NIH grant #CA73056 (SB-E), and by a grant from the Chief Scientist, Israel Ministry of Health (SB-E). We thank Dr Levinson for his assistance. We also thank the subjects for volunteering to participate in the study.

### REFERENCES

Bachen EA, Manuck SB, Cohen S, Muldoon MF, Raible R, Herbert TB and Rabin BS (1995) Adrenergic blockade ameliorates cellular immune responses to mental stress in humans. *Psychosom Med* **57**: 366–372

Ben-Eliyahu S (1998a) Does surgery for the removal of cancer promotes metastasis? Mediating mechanisms and potential Prophylactic Measures (conference abstract). *Neuroimmunomodulation* **5**: 5

Ben-Eliyahu S (1998b) Stress, natural killer cell activity, and tumor metastasis The role of catecholamines and corticosteroids. In: Levi A, Grauer E, Ben-Nathan D and De-Kloet ER (eds) *New Frontiers in Stress Research: Modulation in Brain Function*, pp 203–215. Harwood Academic Publishers GmbH: Amsterdam

Ben-Eliyahu S, Page GG, Shakhar G and Taylor AN (1996) Increased susceptibility to metastasis during pro-oestrus/oestrus in rats: possible role of oestradiol and natural killer cells. *Br J Cancer* **74**: 1900–1907

Ben-Eliyahu S, Page GG, Yirmiya R and Shakhar G (1999) Evidence that stress and surgical interventions promote tumor development by suppressing natural killer cell activity. *Int J Cancer* **80**: 880–888

Ben-Eliyahu S, Shakhar K and Shakhar G (2000) Timing of adrenergic stimulation within the estrous cycle affects resistance to metastasis and levels of NK cell activity: Possible clinical implications. *Br J Cancer* **83**: 1747–1754

Benschop R, Rodriguez-Feuerhahn M and Schedlowski M (1996a) Catecholamine-induced leukocytosis: early observations, current research, and future directions. *Brain Behav Immun* **10**: 77–91

Benschop RJ, Jacobs R, Sommer B, Schurmeyer TH, Raab JR, Schmidt RE and Schedlowski M (1996b) Modulation of the immunologic response to acute stress in humans by beta-blockade or benzodiazepines. *Faseb J* **10**: 517–524

Beynon HL, Garbett ND and Barnes PJ (1988) Severe premenstrual exacerbations of asthma: effect of intramuscular progesterone. *Lancet* **2**: 370–372

Brittenden J, Heys SD, Ross J and Eremin O (1996) Natural killer cells and cancer. *Cancer* **77**: 1226–1243

Case AM and Reid RL (1998) Effects of the menstrual cycle on medical disorders. *Arch Intern Med* **158**: 1405–1412

Cohen S and Herbert TB (1996) Health psychology: psychological factors and physical disease from the perspective of human psychoneuroimmunology. *Annu Rev Psychol* **47**: 113–142

Dengler HG and Hengstmann JH (1976) Metabolism and pharmacokinetics of orciprenaline in various animal species and man. *Arch Int Pharmacodyn Ther* **223**: 71–87

Friberg DD, Bryant JL and Whiteside TL (1996) Measurements of natural killer (NK) activity and NK-Cell quantification. *Methods* **9**: 316–326

Giboney Page G and Ben-Eliyahu S (2000) Natural killer cell activity and resistance to tumor metastasis in prepubescent rats: Deficient baselines, but invulnerability to stress and b-adrenergic stimulation. *NeuroImmunoModulation* **7**: 160–168

Hagen AA and Hrushesky WJ (1998) Menstrual timing of breast cancer surgery. *Am J Surg* **175**: 245–261

Hrushesky WJ, Gruber SA, Sothern RB, Hoffman RA, Lakatua D, Carlson A, Cerra F and Simmons RL (1988) Natural killer cell activity: age, estrous- and circadian-stage dependence and inverse correlation with metastatic potential. *J Natl Cancer Inst* **80**: 1232–1237

Irwin M (1993) Stress-induced immune suppression. Role of the autonomic nervous system. *Ann N Y Acad Sci* **697**: 203–218

Jetschmann JU, Benschop RJ, Jacobs R, Kemper A, Oberbeck R, Schmidt RE and Schedlowski M (1997) Expression and in-vivo modulation of alpha- and beta-adrenoceptors on human natural killer (CD16+) cells. *J Neuroimmunol* **74**: 159–164

Kiecolt-Glaser JK and Glaser R (1995) Psychoneuroimmunology and health consequences: data and shared mechanisms. *Psychosom Med* **57**: 269–274

Klokker M, Secher NH, Madsen P, Pedersen M and Pedersen BK (1997) Adrenergic beta 1- and beta 1 + 2-receptor blockade suppress the natural killer cell response to head-up tilt in humans. *J Appl Physiol* **83**: 1492–1498

Landmann R (1992) Beta-adrenergic receptors in human leukocyte subpopulations. *Eur J Clin Invest* **1**: 30–36

Laudenslager ML, Rasmussen KL, Berman CM, Lilly AA, Shelton SE, Kalin NH and Suomi SJ (1999) A preliminary description of responses of free-ranging rhesus monkeys to brief capture experiences: behavior, endocrine, immune, and health relationships. *Brain Behav Immun* **13**: 124–137

Litschauer B, Zauchner S, Huemer KH and Kafka-Lutzow A (1998) Cardiovascular, endocrine, and receptor measures as related to sex and menstrual cycle phase. *Psychosom Med* **60**: 219–226

Lozzio BB and Lozzio CB (1979) Properties and usefulness of the original K-562 human myelogenous leukemia cell line. *Leuk Res* **3**: 363–370

Manhem K, Jern C, Pilhall M, Shanks G and Jern S (1991) Haemodynamic responses to psychosocial stress during the menstrual cycle. *Clin Sci (Colch)* **81**: 17–22

Manhem K, Hansson L, Milsom I, Pilhall M and Jern S (1996) Estrogen and progestagen modify the hemodynamic response to mental stress in young women. *Acta Obstet Gynecol Scand* **75**: 57–62

Marcus DA (1995) Interrelationships of neurochemicals, estrogen, and recurring headache [see comments]. *Pain* **62**: 129–139

Mills PJ, Ziegler MG, Dimsdale JE and Parry BL (1995) Enumerative immune changes following acute stress: effect of the menstrual cycle. *Brain Behav Immun* **9**: 190–195

Ottenhof PC, Morales A and Baines MG (1981) Quantitation of a whole blood assay for human natural killer cell activity. *J Immuno Methods* **42**: 305–318

Pauli BD, Reid RL, Munt PW, Wigle RD and Forkert L (1989) Influence of the menstrual cycle on airway function in asthmatic and normal subjects. *Am Rev Respir Dis* **140**: 358–362

Pollock RE, Zimmerman SO, Fuchshuber P and Lotzova E (1990) Lytic units reconsidered: pitfalls in calculation and usage. *J Clin Lab Anal* **4**: 274–282

Pollock RE, Lotzova E and Stanford SD (1991) Mechanism of surgical stress impairment of human perioperative natural killer cell cytotoxicity. *Arch Surg* **126**: 338–342

Ree RC and Platts AA (1983) A modified short-term cytotoxicity test: assessment of natural cell mediated cytotoxicity in whole blood. *J Immunol Methods* **62**: 79–83

Santala M, Vilska S, Saarikoski S and Castren O (1990) Lymphocyte beta 2-adrenoceptor density during menstrual cycle and pregnancy, in delivery and puerperium. *Eur J Obstet Gynecol Reprod Biol* **34**: 79–87

Schedlowski M, Jacobs R, Stratmann G, Richter S, Hadicke A, Tewes U, Wagner TO and Schmidt RE (1993) Changes of natural killer cells during acute psychological stress. *J Clin Immunol* **13**: 119–126

Shakhar G and Ben-Eliyahu S (1998) In vivo beta-adrenergic stimulation suppresses natural killer activity and compromises resistance to tumor metastasis in rats. *J Immunol* **160**: 3251–3258

Sheridan JF, Dobbs C, Brown D and Zwilling B (1994) Psychoneuroimmunology: stress effects on pathogenesis and immunity during infection. *Clin Microbiol Rev* **7**: 200–212

Shimizu N, Kaizuka Y, Hori T and Nakane H (1996) Immobilization increases norepinephrine release and reduces NK cytotoxicity in spleen of conscious rat. *Am J Physiol* **271**: R537–544

Sulke A, Jones D and Wood P (1985) Variation in natural killer activity in peripheral blood during the menstrual cycle. *Br Med J (Clin Res Ed)* **290**: 884–886

Taketomi A, Shimada M, Shirabe K, Kajiyama K, Gion T and Sugimachi K (1998) Natural killer cell activity in patients with hepatocellular carcinoma: a new prognostic indicator after hepatectomy. *Cancer* **83**: 58–63

Tan KS, McFarlane LC, Coutie WJ and Lipworth BJ (1996) Effects of exogenous female sex-steroid hormones on lymphocyte beta 2-adrenoceptors in normal females. *Br J Clin Pharmacol* **41**: 414–416

Tersman Z, Collins A and Eneroth P (1991) Cardiovascular responses to psychological and psychological stressors during the menstrual cycle. *Psychosom Med* **53**: 185–197

Thyss A, Caldani C, Bourcier C, Benita G and Schneider M (1984) Comparison of natural killer activity during the first and second halves of the menstrual cycle in women [letter]. *Br J Cancer* **50**: 127–128

Whalen MM and Bankhurst AD (1990) Effects of beta-adrenergic receptor activation, cholera toxin and forskolin on human natural killer cell function. *Biochem J* **272**: 327–331

Wheeldon NM, Newnham DM, Coutie WJ, Peters JA, McDevitt DG and Lipworth BJ (1994) Influence of sex-steroid hormones on the regulation of lymphocyte beta 2-adrenoceptors during the menstrual cycle. *Br J Clin Pharmacol* **37**: 583–588

White D, Jones D, Cooke T and Kirkham N (1982) Natural killer (NK) activity in peripheral blood lymphocytes of patients with benign and malignant breast disease. *Br J Cancer* **46**: 611–616

Yovel G, Shakhar G, Rosenne E, Frenk H and Ben-Eliyahu S (1998) Effects of gender, menstrual cycle, and oral contraceptives on number and activity of human NK cells: a role for serum factor? *Society for Neuroscience Abstracts* **24**: 1854

Zoller M, Heumann U, Betzler M, Stimmel H and Matzku S (1989) Depression of nonadaptive immunity after surgical stress: influence on metastatic spread. *Invasion Metastasis* **9**: 46–68

 *© 2000 Cancer Research Campaign*
